# Truncated Tau Induces Mitochondrial Transport Failure Through the Impairment of TRAK2 Protein and Bioenergetics Decline in Neuronal Cells

**DOI:** 10.3389/fncel.2020.00175

**Published:** 2020-07-30

**Authors:** Rodrigo A. Quintanilla, Carola Tapia-Monsalves, Erick H. Vergara, María José Pérez, Alejandra Aranguiz

**Affiliations:** Laboratory of Neurodegenerative Diseases, Facultad de Ciencias de la Salud, Instituto de Ciencias Biomédicas, Universidad Autónoma de Chile, Santiago, Chile

**Keywords:** kinesin, mitochondria, tau, TRAK2/Milton, transport, truncated tau

## Abstract

Mitochondria are highly specialized organelles essential for the synapse, and their impairment contributes to the neurodegeneration in Alzheimer’s disease (AD). Previously, we studied the role of caspase-3–cleaved tau in mitochondrial dysfunction in AD. In neurons, the presence of this AD-relevant tau form induced mitochondrial fragmentation with a concomitant reduction in the expression of Opa1, a mitochondrial fission regulator. More importantly, we showed that caspase-cleaved tau affects mitochondrial transport, decreasing the number of moving mitochondria in the neuronal processes without affecting their velocity rate. However, the molecular mechanisms involved in these events are unknown. We studied the possible role of motor proteins (kinesin 1 and dynein) and mitochondrial protein adaptors (RhoT1/T2, syntaphilin, and TRAK2) in the mitochondrial transport failure induced by caspase-cleaved tau. We expressed green fluorescent protein (GFP), GFP-full-length, and GPF-caspase-3–cleaved tau proteins in rat hippocampal neurons and immortalized cortical neurons (CN 1.4) and analyzed the expression and localization of these proteins involved in mitochondrial transport regulation. We observed that hippocampal neurons expressing caspase-cleaved tau showed a significant accumulation of a mitochondrial population in the soma. These changes were accompanied by evident mitochondrial bioenergetic deficits, including depolarization, oxidative stress, and a significant reduction in ATP production. More critically, caspase-cleaved tau significantly decreased the expression of TRAK2 in immortalized and primary hippocampal neurons without affecting RhoT1/T2 and syntaphilin levels. Also, when we analyzed the expression of motor proteins—Kinesin 1 (KIF5) and Dynein—we did not detect changes in their expression, localization, and binding to the mitochondria. Interestingly, the expression of truncated tau significantly increases the association of TRAK2 with mitochondria compared with neuronal cells expressing full-length tau. Altogether these results indicate that caspase-cleaved tau may affect mitochondrial transport through the increase of TRAK2–mitochondria binding and reduction of ATP production available for the process of movement of these organelles. These observations are novel and represent a set of exciting findings whereby tau pathology could affect mitochondrial distribution in neurons, an event that may contribute to synaptic failure observed in AD.

## Introduction

Mitochondria are the mastermind organelles in charge of energy production, calcium regulation, and antioxidant defenses (reviewed in Pérez et al., 2018a). Mitochondria contribute to several synaptic processes, including neurotransmitter vesicle recycling, calcium buffering, dendritic spine formation, and neuronal plasticity (Sheng, 2017; Pérez et al., 2018a). Synaptic efficiency requires a perfect distribution and localization of the presynaptic and postsynaptic elements where mitochondria play a vital role (Macaskill and Kittler, 2010; Sheng, 2017).

Several neurodegenerative disorders, like Huntington disease (HD), Parkinson’s disease (PD), and Alzheimer’s disease (AD), showed deficiencies in axonal transport (De Vos et al., 2008). Also, accumulative studies showed several signs of mitochondrial impairment, including defects in bioenergetics, dynamics, and transport in different cells and mice models related to AD (Pérez et al., 2018b). AD is a neurodegenerative disease that is characterized by the formation of extracellular senile plaques and intraneuronal aggregates formed by abnormally modified tau protein (Mucke, 2009; Götz et al., 2019). Tau is a neuronal protein involved in the stabilization of microtubules, and its pathological modifications can also affect neuronal function, including axonal transport (Quinn et al., 2018; Tapia-Rojas et al., 2019). In this context, the genetic reduction of tau expression prevented the impairment of mitochondrial transport induced by the treatment of hippocampal neurons with Aβ peptide (Vossel et al., 2010, 2015). Also, the presence of a pathological tau modification such as hyperphosphorylation reduced mitochondrial transport through the axon (Rodríguez-Martín et al., 2013) by a mechanism that involved the impairment of different motor proteins (Shahpasand et al., 2012). Therefore, these findings indicate that tau could be a vital element involved in the regulation of mitochondrial transport in neurons.

Defects in mitochondrial health induced by pathological modifications of tau can also affect neuronal communication (Quintanilla et al., 2009, 2012, 2014; Manczak and Reddy, 2012; Pérez et al., 2018b). In this context, we studied the effects of caspase 3–cleaved tau, a relevant pathological modification in AD (de Calignon et al., 2010; Ozcelik et al., 2016), on mitochondrial function in neuronal cells. Expression of truncated tau affected mitochondrial health-inducing depolarization and mitochondrial fragmentation through the impairment of the mitochondrial fusion regulator Opa1 (Quintanilla et al., 2012, 2014; Pérez et al., 2018b). More interestingly, the expression of this cleaved tau form reduced mitochondrial movement in hippocampal neurons, suggesting that these actions could be relevant for the synaptic deficiency reported in AD (Quintanilla et al., 2012, 2014).

In neurons, mitochondrial transport is regulated for different cargo and adaptor proteins (Macaskill and Kittler, 2010; Sheng, 2017). Mitochondrial movement between axons and synaptic terminals is produced by the association of these organelles with motor proteins such as kinesin family proteins (KIFs) and dynein (Zinsmaier et al., 2009; Moore and Holzbaur, 2018). Dynein mediates the retrograde microtubular transport of several elements (from axon to soma; Zinsmaier et al., 2009; Sheng, 2017), while kinesin-1 proteins (knows as KIF5) participate in retrograde organelle transport (from neuronal soma to axon; Zinsmaier et al., 2009; Sheng, 2017). Kinesin-1 family members, also known as KIF5A, KIF5B, and KIF5C, are the main motors driving mitochondrial transport in neurons (Zinsmaier et al., 2009; Macaskill and Kittler, 2010; Ari et al., 2014; Sheng, 2017). Kinesin-1 is usually composed of two heavy kinesin chains (KHC), which contain the microtubule and nucleotide-binding domains and two kinesin light chains (KLC), which play a role in binding the cargo (Glater et al., 2006; Stokin and Goldstein, 2006). However, for mitochondrial transport, the KLC is replaced by mitochondrial accessory proteins such as Milton (also called TRAK1/2) and Miro (also called RhoT1/2 GTPases), both of which can interact directly with the KHC domain (Brickley et al., 2005). TRAK1/2 binds to KHC and also interacts with the mitochondrial membrane protein RhoT1/2 (Stowers et al., 2002; Reis et al., 2009; Zinsmaier et al., 2009). This interaction between TRAK1/2/ and RhoT1/2 ((López-Doménech et al., 2018) is essential for the axonal localization of mitochondria and for directing mitochondrial movement to the synaptic terminals according to the neuronal demands (Sheng, 2017). Also, for functional purposes, mitochondria need to remain stationary, while 20–30% of mitochondria are considered motile (Sheng, 2017). Moving mitochondria can also become stationary, and these organelles can be transported again in response to changes in bioenergetic status and synaptic activity (Misgeld and Schwarz, 2017). These actions are coordinated by syntaphilin, a protein that acts as a specific brake for axonal mitochondria (Kang et al., 2008), allowing these organelles to be anchored to microtubules (Misgeld and Schwarz, 2017).

Several reports indicate that axonal transport is impaired in AD, and this dysregulation contributes to neuronal dysfunction (Stokin and Goldstein, 2006; De Vos et al., 2008; Ari et al., 2014). For example, studies in AD brain samples showed that the expression of KIF5A, KIF1B, and KIF21B at gene and protein levels was significantly increased compared with age-matched controls (Hares et al., 2017). Also, KIF5A protein expression levels correlated inversely with the levels of APP and soluble Aβ in AD brains, indicating that these effects may be an adaptive response to impaired axonal transport in AD (Hares et al., 2017). However, it is unclear whether mitochondrial transport accessories proteins such as TRAK1/2, RhoT1/T2, and syntaphilin could be involved in mitochondrial movement deficiencies in the context of AD or any other neurodegenerative diseases.

Interestingly, several studies have shown that tau overexpression inhibits mitochondrial movement in various neuronal cell types (Stoothoff et al., 2009; Vossel et al., 2010). Also, studies by Shahpasand et al. (2012) showed that overexpression of wild-type tau or mutated forms that represent tau hyperphosphorylation in AT8 epitopes (Ser199, Ser202, and Thr205) reduced mitochondrial movement in neuronal cells. More importantly, our studies showed that the expression of caspase-cleaved tau (GFP-T4C3) in hippocampal neurons equally impairs anterograde/retrograde mitochondrial transport, reducing the number of moving mitochondrial elements compared to neurons that expressed full-length tau (Quintanilla et al., 2012, 2014). Even though the overexpression or pathological modifications of tau can affect mitochondrial transport, the mechanism behind these defects is unknown.

**Figure 1:**
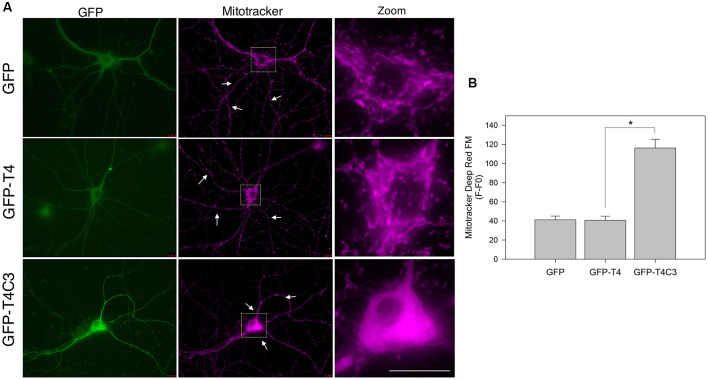
Expression of caspase-3–cleaved tau reduces mitochondrial localization in the neuronal processes of hippocampal neurons. **(A)** Representative images of rat hippocampal neurons transfected with green fluorescent protein (GFP), GFP-T4 (full-length tau), and GFP-T4C3 (caspase-cleaved tau) were loaded with MitoTracker Deep Red FM to determine mitochondrial localization. Arrows indicate isolated mitochondria in neurons. Zoom images shows a magnified were region of neuronal soma showing mitochondrial localization in this area. **(B)** Quantification of fluorescence intensity of MitoTracker Deep Red FM (*F*) per area minus background fluorescence (*F*0), which represents mitochondrial localization in the somatic region of hippocampal neurons. Caspase-cleaved tau increases mitochondrial localization in the soma compared with neurons expressing vector (GFP) or full-length tau (GFP-T4). Data are mean ± standard error (SE), *n* = 3. *p < 0.001 indicates differences between groups calculated by ANOVA test. Bar = 20 μm.

**Figure 2:**
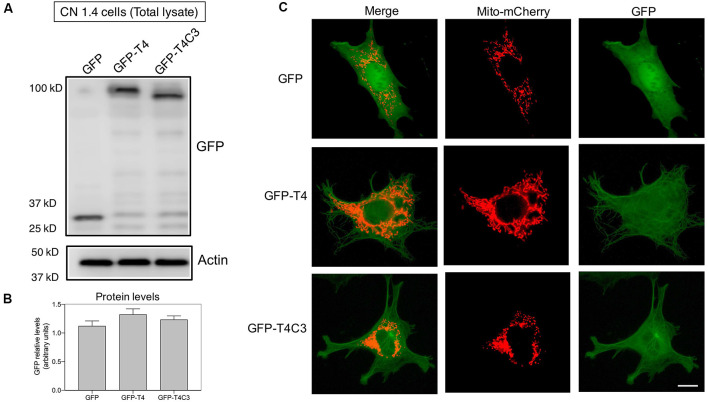
Expression of pathological forms of tau in immortalized cortical neurons. **(A)** CN1.4 cells were transfected with GFP and tau constructs (GFP-T4, GFP-T4C3) to determine the expression of the different forms of tau using GFP by western blot. Whole-cell extracts were collected, and GFP levels were evaluated 48 h after transfection. Representative images show that GFP expression levels are similar, indicating equal expression levels of the different forms of tau. Images also show the levels of β-actin as a loading control. **(B)** Data represent the mean ± SE, *n* = 4. GFP levels were normalized against β-actin levels. **(C)** CN1.4 cells were double transfected with Mito-mCherry (a mitochondrial construct) and the GFP-tau constructs (GFP-T4, GFP-T4C3). Representative fluorescent images were taken in live cells. Bar = 10 μm.

**Figure 3:**
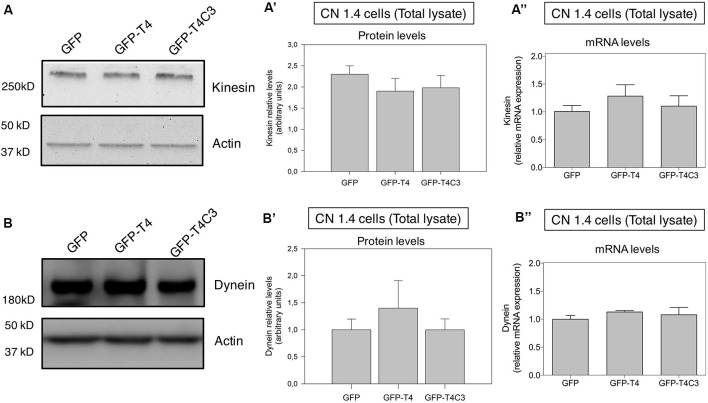
Effects of caspase-cleaved tau on kinesin 1 and dynein expression in immortalized cortical neurons. Panels **(A,B)** show representative western blot images of CN 1.4 cells transfected with GFP, GFP-T4 (normal tau), and GFP-T4C3 (truncated tau) showing the relative kinesin 1 (KIF5) or dynein expression levels. Also, Panels **(A′,B′)** show a quantitative analysis of the relative expression levels of kinesin 1 **(A′)** and dynein **(B′)** in immortalized cells transfected with GFP and GFP-tau (s) forms. Data are mean ± SE, *n* = 3. Total RNA was extracted from immortalized cortical neurons transfected with GFP, GFP-T4 (full-length tau), and GFP-T4C3 (truncated tau), and mRNA expression levels of kinesin **(A″)** and dynein **(B″)** were measured by RT-PCR (Jara et al., 2018). Data represent mean ± SE, *n* = 4.

**Figure 4:**
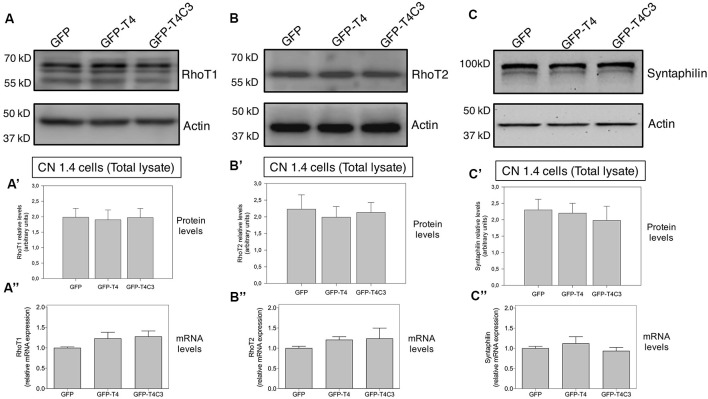
Effects of caspase-cleaved tau on the expression of mitochondrial accessory proteins—RhoT1/T2 and syntaphilin—in immortalized cortical neurons. Panels **(A–C)** show representative western blot images of CN 1.4 cells transfected with GFP, GFP-T4 (normal tau), and GFP-T4C3 (truncated tau), indicating the relative RhoT1 **(A′)**, RhoT2 **(B′)**, and **(C′)** syntaphilin expression levels. Also, Panels **(A′–C′)** show quantitative analysis of the relative expression levels of RhoT1 **(A′)**, RhoT2 **(B′)**, and syntaphilin **(C′)** in immortalized cells transfected with GFP and GFP-tau (s) forms. Data are mean ± SE, *n* = 3. Total RNA was extracted from immortalized cortical neurons transfected with GFP, GFP-T4 (full-length tau), and GFP-T4C3 (truncated tau) and mRNA expression levels of** (A″)** RhoT1, **(B″)** RhoT2, and **(C″)** syntaphilin were measured by RT-PCR (Jara et al., 2018). Data represent mean ± SE, *n* = 4.

**Figure 5:**
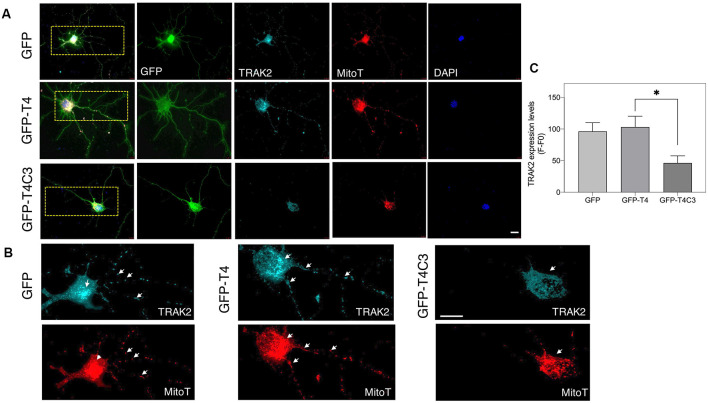
Truncated tau reduces TRAK2 expression and increases its localization in the neuronal soma of hippocampal neurons. **(A)** Representative immunofluorescence images of rat hippocampal neurons transfected with GFP, GFP-T4 (normal tau), and GFP-T4C3 (truncated tau) showing TRAK2 expression and mitochondrial staining (MitoTracker Red CMXRos, a fixable mitochondrial dye). Expression of GFP-T4C3 reduces TRAK2 levels compared with neurons expressing GFP-T4 (normal tau). **(B)** Higher magnification images from **(A)** showing a detail of the TRAK2 localization and expression pattern in hippocampal neurons transfected with the indicated conditions. Cells transfected with caspase-cleaved tau show an apparent reduction in TRAK2 expression and an apparent increase in the localization of TRAK2 with MitoTracker Red CMXRos, suggesting an increase in the association of TRAK2 with mitochondria. White arrows indicate presence of mitochondria in neuronal processes, which is reduced in neurons that expressed caspase-cleaved tau. **(C)** Quantitative analysis of TRAK2 fluorescence levels obtained from hippocampal neurons transfected with GFP, GFP-T4, and GFP-T4C3. Truncated tau reduced TRAK2 expression compared to neurons that expressed full-length tau (GFP-T4). Data are mean ± SE, *n* = 4. ^*^p < 0.05 indicates differences between groups calculated by ANOVA test. Immunofluorescence studies are representative of four independent experiments. Bar = 20 μm.

**Figure 6:**
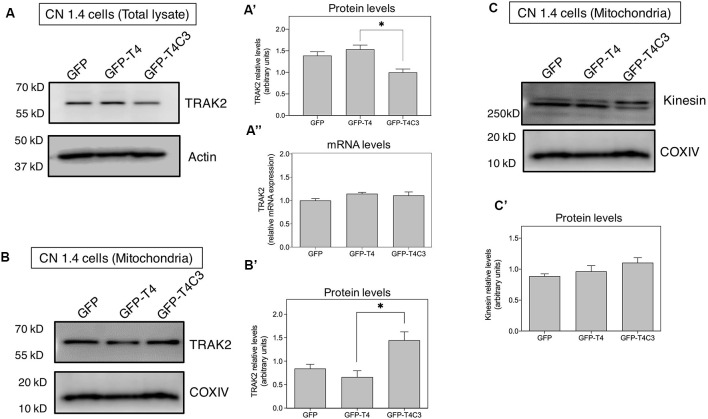
Truncated tau reduces TRAK2 expression and increases its association with mitochondrial fraction in immortalized cortical neurons. **(A)** Representative western blot images of CN 1.4 cells transfected with GFP, GFP-T4 (normal tau), and GFP-T4C3 (truncated tau) indicating the relative TRAK2 expression levels. Caspase-cleaved tau decreases TRAK2 expression compared with cells that were expressing GFP or GFP-T4 (normal tau). **(A′)** Quantitative analysis of the relative expression levels of TRAK2 in immortalized neurons transfected with indicated conditions. Data are mean ± SE, *n* = 5. ^*^p < 0.05 indicates differences between groups calculated by ANOVA test. **(A″)** Total RNA was extracted from immortalized cortical neurons transfected with GFP, GFP-T4 (full-length tau), and GFP-T4C3 (truncated tau) and mRNA expression levels of TRAK2 were measured by RT-PCR. Data represent mean ± SE, *n* = 3. **(B)** Representative western blot images of CN 1.4 cells for mitochondrial extracts transfected with the conditions indicated.** (B′)** Quantitative graph showing that caspase-cleaved tau expression increases TRAK2 presence in mitochondrial extracts compared with cells transfected with the full-length tau form (GFP-T4). Data are mean ± SE, *n* = 4. ^*^p < 0.05 indicates differences between groups calculated by ANOVA test. **(C)** Representative western blot images and quantitative analysis **(C′)** of kinesin 1 protein levels in mitochondrial extracts of CN 1.4 cells transfected with the conditions indicated (*n* = 4).

**Figure 7:**
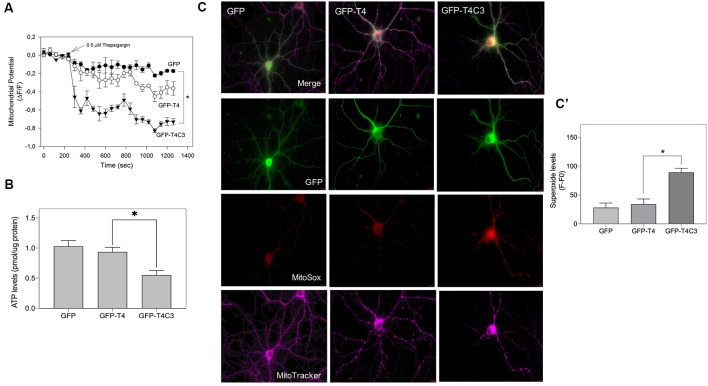
Caspase-3–cleaved tau expression reduces ATP production and mitochondrial membrane potential in immortalized cortical neurons. **(A)** CN 1.4 cells were transfected with the indicated conditions and loaded with MitoTracker red CM-H_2_XRos to evaluate mitochondrial membrane potential levels (Quintanilla et al., 2009, 2012, 2013, 2014; Pérez et al., 2018b). Cells were challenged with thapsigargin (0.5 μM, 30 min) to mobilize a small calcium concentration, and changes in mitochondrial potential were determined by live-cell imaging. Treatment with thapsigargin significantly decreases mitochondrial potential levels in cells transfected with caspase-cleaved tau **(A)**. The graph shows representative plots of mitochondrial potential changes in cells transfected with the indicated conditions. Data represent the mean ± SE, *n* = 4. Statistical differences, *p < 0.05, were estimated using one-way ANOVA. **(B)** Immortalized cortical neurons were transfected with GFP, GFP-T4, and GFP-T4C3, and ATP levels were evaluated using a bioluminescence assay kit (see “Materials and Methods” section). Data represent the mean ± SE, *n* = 3. Statistical differences, ^*^p < 0.05, were estimated using one-way ANOVA. **(C)** Representative fluorescence images of hippocampal neurons transfected with GFP, GFP-T4 (normal tau), and GFP-T4C3 (caspase-cleaved tau) loaded with MitoSox (superoxide indicator) and MitoTracker Deep Red (mitochondrial marker). Caspase-cleaved tau induces a significant increase in superoxide levels compared with cells positive for GFP-T4 (normal tau). **(C′)** Graph bars show a quantitative analysis of MitoSox fluorescence levels obtained from hippocampal neurons transfected with GFP, GFP-T4, and GFP-T4C3. Truncated tau increased superoxide production compared to neurons that expressed full-length tau (GFP-T4). Data are mean ± SE, *n* = 3. *p < 0.05 indicates differences between groups calculated by ANOVA test. Bar = 20 μm.

Therefore, we studied the events behind mitochondrial transport alterations induced by caspase-3–cleaved tau, on the principal components of the transport machinery present in hippocampal neurons (Figures 1–7). Expression of caspase-cleaved tau reduced mitochondrial localization in neuronal processes and induced different bioenergetic defects, including mitochondrial depolarization, increase of reactive oxygen species (ROS), and a decrease in ATP production. We investigated the expression levels of kinesin 1 and dynein in neuronal cells expressing full-length and truncated tau in immortalized cortical neurons with suppressed expression of tau (Bongarzone et al., 1998). Caspase-3–cleaved tau did not affect kinesin 1 and dynein expression and did not have any effects on the expression of mitochondrial accessory motor proteins such as RhoT1/T2 and syntaphilin. Interestingly, caspase-cleaved tau reduced the expression of TRAK2 in hippocampal neurons and immortalized cortical neurons. Finally, we analyzed the levels of TRAK2 and kinesin 1 in separate extracts of cytosol and mitochondria in cortical neurons transfected with green fluorescent protein (GFP) and the tau forms. These studies showed that in cells that expressed caspase-cleaved tau, TRAK2 is more present in the mitochondrial fraction compared with cells expressing full-length tau.

## Materials and Methods

### Cell Culture

Conditionally immortalized neuronal cell lines (CN1.4 cells; Bongarzone et al., 1998; Quintanilla et al., 2009; Pérez et al., 2018b) were cultured in DMEM media (Mediatech CellGro, Corning, NY, USA) supplemented with 5% inactivated fetal bovine serum (Mediatech Inc., Manassas, VA, USA) and 1% penicillin/streptomycin (Mediatech Inc., CellGro, Corning, NY, USA) and incubated at 33°C and 5% CO_2_. These cells were immortalized by retroviral infection of enriched cortical neuronal cultures that adopt a morphology similar to that of cortical neurons and express low endogenous levels of tau (Bongarzone et al., 1998; Krishnamurthy and Johnson, 2004). Hippocampal neuronal cultures were prepared from Sprague–Dawley dam pregnant rats at embryonic day 18, according to Quintanilla et al. (2012, 2014). Pregnant dams were obtained from Bioterio Central, P. Universidad Catolica de Chile, Santiago, Chile. Cultures were maintained in neurobasal growth medium (Thermo Fisher Scientific, Waltham, MA, USA) supplemented with B27 (Thermo Fisher, Scientific), L-glutamine, penicillin, and streptomycin. On day 3, the cultured neurons were treated with cytosine arabinose (Sigma Aldrich, St. Louis, MO, USA) for 72 h to obtain cultures highly enriched in neurons (~5% glia), and on day 6, the cultured neurons were transfected (Quintanilla et al., 2014; Pérez et al., 2018b). All procedures were performed in agreement with the Institutional Bioethics Committee of Universidad Autonoma de Chile (Resolution 0049-17, May 2017).

### Tau Constructs

Tau constructs tagged with GFP, GFP-full-length tau (GFP-T4 in the figures), and GFP-cleaved tau [GFP-T4C3 in the figures; GFP-tau (s)] were generated as previously described (Quintanilla et al., 2009, 2012, 2014). CN1.4 cells and hippocampal neurons were transiently transfected with plasmids containing tau constructs using Lipofectamine 2000 (Thermo Fisher Scientific) diluted in OptiMEM (Thermo Fisher Scientific; Quintanilla et al., 2012, 2014; Pérez et al., 2018b). Complementary CN 1.4 cells transfected with GFP-related constructs were transfected with Mito-mCherry (Pérez et al., 2018b) to evaluate mitochondrial morphology (Figure 2C). Media were changed at 24 h posttransfection, and analyses were conducted 48 h posttransfection in CN 1.4 cells and 15 days *in vitro* for studies in primary hippocampal neurons. The GFP plasmid expression was verified with live-cell imaging, observing a 40% and 8% of transfection efficiency in CN 1.4 and primary neurons, respectively (Pérez et al., 2018b). Also, GFP, GFP-full-length tau (GFP-T4), and GFP-truncated tau (GFP-T4C3) expression levels were estimated in CN 1.4 cells by detecting GFP expression using western blot (Figure 2).

### Western Blot Analysis

CN1.4 cells were lysed in Triton lysis buffer, including a protease inhibitor cocktail (Roche Applied Science, Mannheim, Germany) and a phosphatase inhibitor (Jara et al., 2018; Pérez et al., 2018b). Thirty micrograms of total protein extracts were resolved by electrophoresis on a sodium dodecyl sulfate (SDS)-polyacrylamide gel and transferred to PVDF membranes (Jara et al., 2018; Pérez et al., 2018b). Membranes were incubated with primary antibodies: polyclonal anti-KIF5A (1:500, Cat. No. PA1-642, Thermo Fisher Scientific), polyclonal anti-dynein (1:500, Cat. No. SC-514579, Santa Cruz Biotechnology, Dallas, TX, USA), polyclonal anti-RhoT1 (1:500, Cat. No. SC-398520, Santa Cruz Biotechnology), polyclonal anti-RhoT2 (1:500, Cat. No. SC-398521, Santa Cruz Biotechnology), polyclonal anti-syntaphilin (1:1,000, Cat. No. PA5-88474, Thermo Fisher Scientific), polyclonal anti-TRAK2 (1:1,000, Cat. No. PA5-21858, Thermo Fisher Scientific), monoclonal anti-COX4 (1:500, Cat. No. SC-376731, Santa Cruz Biotechnology), and monoclonal anti-VDAC (1:500, Cat. No. SC-390996, Santa Cruz Biotechnology) overnight. The equal loading and transfer of protein to the membranes were determined by reprobing with a monoclonal anti-β-actin antibody (1:2,000, Cat. No. SC-8432, Santa Cruz Biotechnology). Protein signal was detected using a horseradish peroxide (HRP)-linked goat anti-mouse or anti-rabbit secondary antibody (1:2,000, Thermo Fisher Scientific), as indicated. Finally, the immunoreactivity of the protein signal was detected using enhanced chemiluminescence reagent (ECL, Thermo Fisher Scientific; Jara et al., 2018; Pérez et al., 2018b). Expression levels of the indicated proteins were estimated related to the intensity of housekeeping protein signals using ImageJ software (NIH, Bethesda, MD, USA).

### Preparation of Mitochondrial Extracts

Transfected CN 1.4 cells with GFP or GFP-tau forms (GFP-full-length tau and GFP-caspase-cleaved tau) were suspended in MSH buffer (230 mM mannitol, 70 mM sucrose, 5 mM HEPES, pH 7.4) supplemented with 1 mM EDTA and protease inhibitor cocktail. Homogenates were centrifuged at 600 *g* for 10 min at 4°C to discard nuclei and cell debris (Jara et al., 2018). The supernatant was centrifuged at 8,000 *g* for 10 min; the new mitochondrial pellet was washed twice in MSH without EDTA. Protein concentration was determined using a standard BCA kit (Thermo Fisher Scientific), and western blot was made accordingly (Jara et al., 2018).

### Mitochondrial Localization and Membrane Potential Determinations

Mitochondrial localization was evaluated in hippocampal neurons expressing GFP, GFP-full-length tau (GFP-T4), and GFP-caspase-cleaved tau (GFP-T4C3) using MitoTracker Deep Red FM (Molecular Probes, Thermo Fisher Scientific) dye according to Quintanilla et al. (2013, 2014), with modifications. Hippocampal neurons were incubated with this dye for 35 min in Krebs–Ringer–HEPES (KRH)–glucose buffer and then were photographed in a high-resolution fluorescence microscope (LX6000 X, Leica; Quintanilla et al., 2012, 2013, 2014). Mitochondrial staining was expressed as the average of the fluorescence signal (*F*) per area in every image (30 images per condition and experiment) minus the intensity of background fluorescence (*F*0). The mitochondrial potential was determined using the mitochondrial dye MitoTracker Red-H_2_XRos (Molecular Probes, Thermo Fisher Scientific), as previously described (Quintanilla et al., 2009, 2012, 2013, 2014; Pérez et al., 2018b). Mitochondrial potential levels were expressed as MitoTracker Red-H_2_XRos fluorescence and are presented as the pseudo ratio (Δ*F*/*F*0), where *F* is the average of the fluorescence signal (*F*) per area in every image and (*F*0) represents the intensity of background fluorescence. Cells were incubated with the dye (100 nM) in KRH–glucose buffer at 37°C for 45 min, and then cells were treated with thapsigargin (0.5 μM, Tocris Bioscience, Minneapolis, MN, USA) for 30 min. The intensity of the signal was analyzed in 10 cells per experiment, in three different studies using ImageJ software (Quintanilla et al., 2009, 2012, 2013, 2014; Pérez et al., 2018b).

### Determination of ROS (Superoxide) Levels

Superoxide levels were evaluated with MitoSox™ (Molecular Probes, Thermo Fisher Scientific) and the mitochondrial dye MitoTracker Deep Red FM (Pérez et al., 2020). Transfected cultured neurons were incubated with 0.5 * μ*M MitoSox and 0.3 * μ*M MitoTracker Deep Red in KRH*–*glucose buffer at 37°C for 30 min (Quintanilla et al., 2013; Pérez et al., 2020). For superoxide determinations with cell imaging, fluorescence images were acquired using the same exposure time and gain to minimize dye photo-bleaching (Quintanilla et al., 2013; Pérez et al., 2020). MitoSox fluorescence (*F*) was determined in each cell, and background (*F*0) of each image was subtracted before normalization per cell area. Normalization was made to ameliorate differences of the dye distribution inside of the cells that present differences in neuronal morphology. Fluorescence images were acquired by live epifluorescence microscopy (Leica LX6000, Wetzlar, Germany) using a 63× oil-immersion objective.

### Immunofluorescence Studies

Hippocampal neurons transfected with GFP or GFP-tau (s) forms were incubated with MitoTracker Red CMXRos (fixable mitochondrial dye, Molecular Probes, Thermo Fisher Scientific) for 35 min in KRH–glucose to evaluate mitochondrial localization. Later, neurons were fixed in 4% paraformaldehyde-KRH for 15 min at 37°C (Quintanilla et al., 2013; Pérez et al., 2018b). Hippocampal cultured neurons were blocked with 10% bovine serum KRH for 1 h and incubated with a primary polyclonal anti-TRAK2 (1:200, Cat. No. PA5-21858, Thermo Fisher Scientific) antibody overnight. After that, the cells were washed in KRH–glucose, probed with the goat secondary polyclonal antibody Alexa Fluor 647 (1:1,000, Cat. No. A32733, Molecular Probes, Thermo Fisher Scientific) for 3 h, and then stained with DAPI (1:5,000, Sigma Aldrich) for 15 min. Estimation of TRAK2 expression in hippocampal neurons was calculated in the neuronal soma, obtaining fluorescence intensity (*F*) and background levels (*F*0) from 25 images (1 or 2 neurons per image) per experiment (total, *n* = 3) using ImageJ software (NIH).

### Determination of ATP Levels

Total ATP levels were measured in immortalized cortical neurons transfected with GFP, GFP-T4 (full-length tau), and GFP-T4C3 (truncated tau) lysates using a luciferin/luciferase bioluminescence assay kit (ATP Determination Kit No. A22066, Molecular Probes, Invitrogen; Jara et al., 2018). The amount of ATP in each sample was calculated from standard curves and normalized to total protein concentration (Jara et al., 2018).

### Real-Time Polymerase Chain Reaction

Dynein, RhoT1/T2, syntaphilin, kinesin, and TRAK2 mRNA levels were analyzed by real-time polymerase chain reaction (RT-PCR). Total RNA was isolated from 100 mg of CN 1.4 lysates transfected with GFP, GFP-T4, and GFP-T4C3 using the TRIzol reagent (Life Technologies, Thermo Fisher Scientific) following the manufacturer’s instructions (Jara et al., 2018). Residual genomic DNA was removed with amplification grade RNase free DNase I (Invitrogen, Thermo Fisher Scientific). RNA yield and purity were measured in a microplate reader (TECAN, Infinite 200 PRO series). One microgram of total RNA was reverse transcribed using the ImProm-II Reverse Transcription System (Promega, Madison, WI, USA) to obtain cDNA, which was stored at –20°C for future use. For quantitative PCR (qPCR) analysis, each cDNA sample was diluted 10× in nuclease-free water. RT-PCR was performed in triplicate in a LightCycler 96 System (Roche Diagnostics GmbH, Roche Applied Science, Mannheim, Germany) using KAPA SYBR FAST qPCR Master Mix (2×) in a final reaction volume of 10 μl (Jara et al., 2018). Amplification conditions consisted of an initial hot start at 95°C for 10 min followed by amplification for 40 cycles (95°C for 15 s, *T*_m_°C for 20 s, and 72°C for 20 s). Melting curves were analyzed immediately after amplification from 55 to 95°C. Expression values were normalized to 18S rRNA levels using the Δ*C*_t_ method. The following primers were used in RT-PCR:

**Table:** 

	FW	RW	*T*_m_
Kinesin 5A	AGGCCAAGCTTTTCCCTCTC	CTGCAGCTACCTGAAAGTGC	58°C
Dynein	GGGTGGACCAGGTACAGTTG	GATGAGCTGCTCGCACTACT	58°C
RhoT1	AGGCCTAATAGATGGCTGTGTTT	GGCCACTTTCCACCTCTCCA	58°C
RhoT2	ACCACCTGCAGAGTCAGGAA	CTAGTGGTTGGGCTAAGGCA	58°C
Syntaphilin	GCCCTCCACCTCTCCCTC	GCGAAGGCTTCCATGGTTTG	58°C
TRAK2	GGGTGGACCAGGTACAGTTG	GATGAGCTGCTCGCACTACT	57°C
GAPDH	GAGTCAACGGATTTGGTCGT	TTGATTTTGGAGGGATCTCG	62°C

*GAPDH, glyceraldehyde 3-phosphate dehydrogenase*.

### Statistical Analysis

For statistical analysis, one-way ANOVA and Dunn’s *a posteriori* test were calculated with SigmaPlot software. Differences were considered significant if *p* < 0.05 or *p* < 0.001, as indicated.

## Results

### Caspase-Cleaved Tau Expression Reduces the Mitochondrial Population in Neuronal Processes

Previously our group studied the role of pathological forms of tau on mitochondrial function and its consequences against neuronal viability (Quintanilla et al., 2009, 2012, 2014; Pérez et al., 2018b). We showed that the expression of caspase-3*–*cleaved tau at D421 (truncated tau) and hyperphosphorylated tau at PHF1 epitopes (S396, S404) affects mitochondrial membrane potential and increases ROS production (Quintanilla et al., 2009, 2012, 2014). More importantly, neurons expressing caspase-cleaved tau showed a significant defect in mitochondrial transport (Quintanilla et al., 2012, 2014). Interestingly, truncated tau decreases the number of moving mitochondria in neuronal processes without affecting the velocity rate of individual mitochondria (Quintanilla et al., 2012, 2014). However, the mechanism involved in these alterations remains unexplored. To address this question, we first analyzed the distribution of a mitochondrial population in hippocampal rat neurons transfected with GFP, GFP-full-length (GFP-T4), and GFP-truncated tau at D421 (GFP-T4C3) using MitoTracker Deep Red FM dye (Figure 1). We observed that expression of truncated tau reduced the number of mitochondria present in neuronal processes compared with neurons transfected with full-length tau (GFP-T4) and GFP (vector; Figure 1, white arrows). We observed that caspase-cleaved tau significantly increases mitochondrial accumulation in the soma of hippocampal neurons compared to cells transfected with full-length tau (Figure 1A, Zoom). Quantification of MitoTracker Deep Red FM fluorescence indicated that truncated tau increases somatic mitochondrial localization compared with the presence of these organelles in neuronal processes shown in neurons transfected with full-length tau and GFP (Figure 1B). These studies indicate that caspase-cleaved tau affects mitochondrial localization in hippocampal neurons, which is in agreement with our previous findings that showed significant alterations in the number of moving mitochondrial elements in neurons expressing truncated tau (Quintanilla et al., 2012, 2014).

### Caspase-Cleaved Tau Expression Does Not Modify the Expression of Kinesin 1 and Dynein Motor Proteins in Immortalized Cortical Neurons

Neurons are polarized cells with axons and neuronal processes that extend a great distance to establish neuronal synapses (Stokin and Goldstein, 2006). This process requires a constant supply of energy, and for that, mitochondrial transport and localization are essential (Zinsmaier et al., 2009). The constant process of anterograde/retrograde mitochondrial transport between axon and dendritic synaptic terminals is produced by association with motor proteins: kinesin family proteins (KIFs) and dynein with microtubular structures (Moore and Holzbaur, 2018). Deficiencies in the expression or mutations in both motor protein components have shown defects in mitochondrial transport, reducing mitochondrial localization in axons (Ari et al., 2014; Hares et al., 2017; Chaudhary et al., 2018). More importantly, we showed that the expression of caspase-cleaved tau equally decreases anterograde/retrograde mitochondrial movement in hippocampal neurons compared with cells expressing full-length tau (Quintanilla et al., 2014). Therefore, we expressed GFP, full-length (GFP-T4), and caspase-3*–*cleaved tau (GFP-T4C3; Figures 2A,B) in CN 1.4 cells (Bongarzone et al., 1998; Ding et al., 2006), an immortalized cortical neuronal model with a minor endogenous expression of tau (Figure 2). Transfection of GFP-related constructs induced a similar expression of GFP and GFP-full-length and GFP-truncated tau levels in immortalized cortical neurons (Figures 2A,B). Also, we expressed GFP and GFP-tau (s) forms and Mito-mCherry in CN 1.4 cells (Figure 2C). The expressions of GFP and GFP-tau (s) forms were very similar and cells expressing truncated tau showed a significant degree of mitochondrial fragmentation, corroborating our previous findings (Quintanilla et al., 2009; Pérez et al., 2018b). Further, we studied kinesin 1 and dynein expression levels in immortalized cortical neurons transfected with GFP, GFP-full-length tau (GFP-T4), and GFP-truncated tau (GFP-T4C3) forms by western blot (Figure 3). We observed that expression of caspase-cleaved tau did not affect the protein levels of kinesin 1 (Figures 3A,A′) or dynein (Figures 3B,B′) compared with cells that expressed GFP and GFP full-length tau (Figures 3A′,B′). Also, truncated tau expression did not affect mRNA expression of kinesin 1 (Figure 3A″) and dynein (Figure 3B″) compared with immortalized cortical neurons transfected with full-length tau.

### Caspase-Cleaved Tau Expression Does Not Modify RhoT1/T2 (Miro1/2) Protein Levels in Immortalized Cortical Neurons

Mitochondrial motor proteins kinesin (KIF5) and dynein are regularly associated with motor adaptor proteins and mitochondrial membrane receptors (Reis et al., 2009; Nemani et al., 2018). These complexes contribute to mitochondrial trafficking and the precise regulation of the distribution of mitochondria in response to different changes in neuronal function (Sheng, 2017). In this context, Miro (know as RhoT1/T2) is a member of the mitochondrial outer membrane Rho GTPase family that acts as a mitochondrial receptor and binds with the motor adaptor protein Milton (or TRAK1/2; Reis et al., 2009; Nemani et al., 2018). In the same context, some studies have shown that dMiro mutant impairs anterograde mitochondrial transport depleting axonal and synaptic mitochondria (López-Doménech et al., 2016). Therefore, in our model, we studied the levels of RhoT1/T2 in immortalized cortical neurons transfected with GFP, full-length, and caspase-cleaved tau (Figures 4A,B). These studies showed that caspase-cleaved tau did not affect RhoT1/T2 protein expression (Figures 4A,A′,B,B′) and mRNA levels (Figures 4A″,B″), indicating that mitochondrial transport impairment is likely not caused by dysfunction of these proteins.

### Caspase-Cleaved Tau Does Not Affect the Mitochondrial Adaptor Syntaphilin in Neuronal Cells

To investigate how truncated tau could affect mitochondrial transport in hippocampal neurons, we studied the expression of syntaphilin, which is a mitochondrial docking protein that contributes to specifically immobilizing mitochondria in axons (Kang et al., 2008; Chen et al., 2009). Syntaphilin is a mitochondrial outer membrane protein whose function is binding mitochondria to microtubules to reduce mitochondrial movement in axons (Kang et al., 2008; Chen et al., 2009). Interestingly, several studies have shown that reducing syntaphilin expression increases axonal mitochondria movement, therefore reducing mitochondrial density within the axons (Lin et al., 2017). We analyzed the expression of syntaphilin in immortalized cortical neuronal cells in response to GFP, full-length, and caspase-cleaved tau expression (Figure 4C). Complementary western blot analysis (Figures 4C,C′) and qPCR analyses (Figure 4C″) did not show any significant changes in syntaphilin protein (Figure 4C″) and mRNA levels (Figure 4C″) in any condition suggesting that accumulation of mitochondria in neuronal soma is not influenced by syntaphilin.

### Truncated Tau Affects the Expression and Localization of the Mitochondrial Adaptor Protein TRAK2 in Neuronal Cells

Milton (as is known in *Drosophila*) is an adaptor protein that binds the mitochondria receptor RhoT1/T2 (Miro1/2; López-Doménech et al., 2018) to kinesin (KIF5; van Spronsen et al., 2013; Loss and Stephenson, 2017). It is known that Milton has two mammalian orthologues, TRAK1 and TRAK2, whose function is vital for mitochondrial localization at synaptic terminals (van Spronsen et al., 2013; Loss and Stephenson, 2017). In the same context, increased expression of TRAK2 increases mitochondrial movement, and its genetic ablation reduces mitochondrial localization in neuronal processes (van Spronsen et al., 2013). Therefore, we studied TRAK2 expression in hippocampal neurons transfected with full-length (GFP-T4) and caspase-cleaved tau (GFP-T4C3) using immunofluorescence methods (Figure 5). We observed a significant reduction in TRAK2 expression in neurons expressing caspase-cleaved tau compared with neuronal cells transfected with normal tau (Figure 5A, white arrows, Figures 5B,C). Quantification of immunofluorescence staining of TRAK2 in hippocampal neurons showed a significant decrease in TRAK2 expression compared to neurons with GFP-full-length tau or GFP (vector) expression (Figure 5C). Also, we complemented immunofluorescence studies using a fixable mitochondrial marker, MitoTracker Red CMXRos, to observe mitochondrial localization (Quintanilla et al., 2013). Interestingly, we observed that truncated tau (GFP-T4C3) expression induced a change in TRAK2 distribution, increasing its accumulation in neuronal soma compared to neurons transfected with GFP or GFP-full-length tau (Figure 5B, white arrows). Complementary TRAK2 expression was evaluated by western blot (Figures 6A,A′) and qPCR analyses (Figure 6A″) in immortalized cortical neurons transfected with GFP, full-length, or caspase-cleaved tau. Truncated tau significantly reduced TRAK2 protein expression levels compared to cells expressing GFP or GFP-full-length tau (Figures 6A,A′). Interestingly, truncated tau expression did not affect mRNA levels of TRAK2 in immortalized cortical neurons (Figure 6A″), indicating that this tau form could be affecting TRAK2 through the modification of a posttranslational mechanism. These studies showed that truncated tau reduced TRAK2 expression levels in immortalized cortical neurons, corroborating our observations in hippocampal neurons (Figures 5A,C).

### Caspase-Cleaved Tau Increases Mitochondrial Localization of TRAK2 in Immortalized Cortical Neurons

Previously, we showed that caspase-cleaved tau reduced TRAK2 expression (Figures 5, 6A,A′) and increased its localization in the neuronal soma (Figure 5B). To study if truncated tau expression affects TRAK2 distribution in neuronal cells, we transfected GFP, full-length (GFP-T4), and caspase-cleaved tau (GFP-T4C3) in immortalized cortical neurons and studied TRAK2 levels in cytosol and mitochondrial fractions (Figures 6A,B). Total and mitochondrial extracts were separated, and we determined TRAK2 expression levels by western blot (Figures 6A,B). These studies showed that the expression of caspase-cleaved tau increased TRAK2 localization with mitochondria, an effect that is evident in western blot assay from mitochondrial extracts (Figures 6B,B′). As a motor protein, kinesin 1 is responsible for mitochondrial transport, and it must be correctly associated with TRAK2 and then with mitochondria to generate movement of mitochondria through microtubules (Sheng, 2017). In this context, we evaluated kinesin 1 localization in the mitochondrial extracts in immortalized cortical neurons transfected with GFP and GFP-tau (s) forms (Figures 6C,C′). Interestingly, we observed that neuronal cells expressing caspase-cleaved tau showed no differences in kinesin 1 levels (KIF 5) in mitochondria preparations compared with neuronal cells transfected with full-length tau (Figure 6C′). These results indicate that the presence of caspase-cleaved tau modifies the TRAK2 interaction with mitochondria, and this effect could be responsible for the accumulation of mitochondria in neuronal soma.

### Expression of Truncated Tau Induces Mitochondrial Depolarization, ATP Reduction, and ROS Increase in Neuronal Cells

Mitochondrial bioenergetics, which includes ROS production, membrane potential, and ATP production, are an essential element for the movement of mitochondria (Pérez et al., 2018a). Therefore, we studied mitochondrial membrane potential levels, ATP production, and ROS levels in immortalized cortical cells and hippocampal neurons transfected with truncated and full-length tau (Figure 7). The mitochondrial potential was estimated using MitoTracker Red *in situ* in transfected cells treated with thapsigargin (0.5 μ, 30 min; Figure 7A). Thapsigargin treatment mildly reduced mitochondrial potential levels in cells transfected with GFP and GFP-full-length tau (Figure 7A). However, truncated tau expression strongly decreased mitochondrial potential in CN 1.4 cells exposed to thapsigargin, indicating that this tau form significantly affects mitochondrial health (Figure 7A). In the same context, we evaluated ATP production in immortalized cortical neurons expressing GFP, GFP-full-length, and GFP-truncated tau (Figure 7B). Interestingly, we observed that truncated tau significantly reduced ATP production compared with cells that expressed full-length tau (Figure 7B). Complementarily, we evaluated superoxide levels in hippocampal neurons transfected with GFP, normal (GFP-T4), and caspase-cleaved tau (GFP-T4C3) using MitoSox dye in live-cell imaging (Figure 7C). Expression of caspase-cleaved tau (GFP-T4C3) increased superoxide levels (Figure 7C) compared to hippocampal neurons expressing full-length tau (GFP-T4; Figure 7C′). Therefore, these results indicate that the presence of truncated tau affects mitochondrial bioenergetics, events that could contribute to the reduction in mitochondrial movement in neurons (Shidara and Hollenbeck, 2010; Watters et al., 2020).

## Discussion

Despite the recognized importance of mitochondria in the synapse, there are still unknown aspects regarding the mechanism of how mitochondrial health affected by the presence of pathological forms of tau contributes to synaptic and cognitive alterations in AD (Pérez et al., 2018a). In this context, the study of mitochondrial transport alterations produced by pathological forms of tau is a pivotal aspect. Tau is a microtubule-associated protein (MAP) that interacts with microtubules, and nonphysiological modifications of tau could cause a destabilization of microtubular elements contributing to neurodegeneration in AD (Tapia-Rojas et al., 2019). Appropriate localization of mitochondria to the synapse is required due to the high demand for ATP production and Ca^2+^ buffering (Macaskill and Kittler, 2010; Sheng, 2017). Previously, we showed that expression of caspase-cleaved tau at D421 by caspase 3 affects mitochondrial transport in hippocampal and cortical cultured neurons (Quintanilla et al., 2012, 2014). Truncated tau reduced the number of moving mitochondria at retrograde and anterograde directions compared with neurons expressing full-length tau (Quintanilla et al., 2014). Here, we complement these observations with our novel findings suggesting an essential role of mitochondria-associated transport accessory protein, TRAK2, in the impairment of movement of mitochondria produced by truncated tau. The expression of this tau form increased the accumulation of mitochondria in the soma, reducing the mitochondrial population in the neuronal processes, including the axon (Figure 1). Using an immortalized cortical neuronal model that does not express tau (Bongarzone et al., 1998; Quintanilla et al., 2009), we studied the effects that full-length and truncated tau could have on the expression of the motor proteins kinesin 1 and dynein, and mitochondria motor accessory proteins RhoT1/T2, synthaphilin, and TRAK2. Truncated tau did not affect the expression of major motor proteins (kinesin 1 and dynein) and also RhoT1/T2 and syntaphilin (Figures 3, 4).

Interestingly, truncated tau significantly reduced TRAK2 expression (Figures 5, 6A) and increased its association with the mitochondria (Figure 6B) compared with cells expressing full-length tau (Figure 6B′). Also, truncated tau expression did not significantly affect kinesin 1 association with mitochondria compared with cells expressing full-length tau and GFP alone (Figures 6C,C′). These observations are related to our previous results that showed that truncated tau expression affects mitochondrial movement equally in the retrograde and anterograde directions, suggesting that defects in kinesin 1 or dynein function may not be involved (Quintanilla et al., 2014). Therefore, it is possible that the defects in the movement of mitochondria could be caused by an apparent malfunction of the mitochondrial adaptor protein TRAK2 induced by truncated tau (van Spronsen et al., 2013).

In different neuronal cell cultures where tau is highly overexpressed (Dixit et al., 2008; Stoothoff et al., 2009; Stamer et al., 2002) or in entirely *in vitro* systems (Morfini et al., 2007), tau can inhibit axonal transport by directly binding to microtubules and preventing the movement of the anterograde motor proteins (Morfini et al., 2007). However, other studies showed clear evidence to the contrary (Yuan et al., 2008; LaPointe et al., 2009). For example, in the squid axoplasm model, the addition of tau at concentrations approximately 20-fold higher than physiological levels did not affect fast axonal transport (Yuan et al., 2008). However, in the same model, a tau construct of only the amino-terminal portion of tau (without the microtubule-binding domain) inhibited transport, as did tau in a conformation that exposed the amino terminus (Götz et al., 2006). These data suggest that anomalous modification of tau may selectively impair axonal transport of mitochondria (Götz et al., 2006) rather than creating a “general roadblock” effect at the level of the microtubules (Miller and Sheetz, 2004; Götz et al., 2006; Ittner et al., 2008). Also, mitochondria with lower mitochondrial membrane potential and bioenergetic impairment are not transported efficiently in the anterograde direction (kinesin-dependent transport; Miller and Sheetz, 2004). Interestingly, we found that caspase-cleaved tau expression affects mitochondrial bioenergetics inducing depolarization, ROS increase, and ATP reduction (Figure 7). Therefore, these mitochondrial defects could contribute to the deficits in mitochondrial transport produced by truncated tau expression.

Mitochondrial transport is actively regulated by local increases in calcium concentration (Yi et al., 2004). Mitochondrial arrest at the synaptic terminal occurs in order for mitochondria to be recruited near the synaptic vesicles, which require energy for release of neurotransmitters (Zinsmaier et al., 2009). Reduction in syntaphilin expression significantly increases the number of mitochondria moving through the axon, improving synaptic activity (Kang et al., 2008). Previously, we showed that truncated tau expression affects mitochondrial bioenergetics (Figure 7) and mitochondrial calcium capacity (Quintanilla et al., 2009). However, in this work, we did not detect significant changes in syntaphilin expression (protein and mRNA levels) in immortalized cortical neurons transfected with GFP and GFP-tau forms (Figures 4C″).

An interesting observation is that the expression of truncated tau increases the association of TRAK2 with mitochondria compared with hippocampal neurons and immortalized cortical neurons transfected with full-length tau (Figures 6B,B′). Also, in mitochondrial extracts, the presence of kinesin 1 or dynein (data not shown) was not affected in neuronal cells expressing truncated tau (Figures 6C,C′). Therefore, the fibrogenic and destabilizing effect of truncated tau on microtubule structures (Ding et al., 2006) could increase TRAK2 attachment to the mitochondria, producing adverse effects against mitochondrial transport. Also, this increased interaction between TRAK2 and mitochondria induced by caspase-cleaved tau could be facilitated by the reduction of ATP production, and mitochondrial depolarization was observed in cells that express this tau form (Figure 7B).

Tau family MAPs also reportedly inhibit kinesin-dependent transport along microtubules (Vershinin et al., 2007; Shigematsu et al., 2018). This inhibition is primarily caused by direct competition between MAPs and kinesin for microtubule binding, reducing the attachment frequency of kinesin (Trinczek et al., 1999). Interestingly, recent work from Shigematsu et al. (2018) studied the ability of tau family MAPs 4R- and 5R-MAP4 [tau isoforms containing (3R) or five (5R) tubulin-binding domains] to form a complex with kinesin-1 and microtubules using cryo-EM studies. These studies showed that both modifications coexist with kinesin-1 on the microtubules and produce an inhibitory effect of MAP4 on kinesin-dependent transport along the microtubules (Shigematsu et al., 2018). However, our present observations indicate that the association between kinesin-1 and mitochondria is not affected by caspase-cleaved tau, which correlates with our previous observations that the direction of mitochondrial movement (anterograde/retrograde) was equally affected by truncated tau expression in hippocampal neurons (Quintanilla et al., 2014). Importantly, these actions could be augmented by the adverse effects that caspase-cleaved tau induces against mitochondria health (Quintanilla et al., 2009, 2012, 2014; Pérez et al., 2018b) and ATP production (Figure 7B). These combinatory actions may reduce mitochondrial localization in neuronal processes, compromising energy supply and calcium regulation for the synaptic process (Macaskill and Kittler, 2010).

Finally, our findings show for the first time that pathological forms of tau can affect mitochondrial transport, enhancing the association of TRAK2 with mitochondria (Figures 6B,B′) and reducing ATP production (Figure 7B). Recent studies have shown that TRAK2 contributes to both axonal and dendritic mitochondrial transport, since TRAK2-shRNA inhibited mobility in these processes in cortical and hippocampal neurons (Brickley and Stephenson, 2011; Loss and Stephenson, 2017). Therefore, it is possible that in our model, a decrease in TRAK2 expression induced by caspase-cleaved tau could be an essential contributor to the reduction in mitochondrial movement in hippocampal neurons. TRAK2 cooperatively binds to kinesin 1 (KIF5; Brickley et al., 2005) and RhoT1/T2 (Loss and Stephenson, 2017) in order to facilitate mitochondrial transport (Reis et al., 2009); however, our evidence indicates that kinesin 1 is likely not involved in the (cannot rule out changes in functional properties by posttransational modifications) impairment of mitochondrial movement (Quintanilla et al., 2014). In the same context, it is possible that caspase-cleaved tau could also affect the binding or interaction of RhoT1/T2 with TRAK2 or with mitochondria to facilitate their movement. While we did not explore this possibility in this work, it could certainly be interesting to test it. Nevertheless, this possibility does not reduce the importance of our finding that pathological modifications of tau could affect mitochondrial movement by modifying the function of TRAK2 and the bioenergetic status of mitochondria.

## Data Availability Statement

The datasets generated for this study are available on request to the corresponding author.

## Ethics Statement

The animal study was reviewed and approved by the Bioethics Committee of the Universidad Autónoma de Chile, Santiago, Chile (Resolution 0049-17, May 2017).

## Author Contributions

RQ conceived the study, performed some experiments, analyzed the data, and wrote the manuscript. CT-M performed some experiments and analyzed data. EV, MP, and AA performed the experiments. All authors read and approved the final version of the manuscript.

## Conflict of Interest

The authors declare that the research was conducted in the absence of any commercial or financial relationships that could be construed as a potential conflict of interest.
